# Altered Brain Functional Connectivity Density in Fast-Ball Sports Athletes With Early Stage of Motor Training

**DOI:** 10.3389/fpsyg.2020.530122

**Published:** 2020-09-25

**Authors:** Chengbo Yang, Ning Luo, Minfeng Liang, Sihong Zhou, Qian Yu, Jiabao Zhang, Mu Zhang, Jingpu Guo, Hu Wang, Jiali Yu, Qian Cui, Huafu Chen, Qing Gao

**Affiliations:** ^1^The Third Department of Physical Education and Training, Chengdu Sport University, Chengdu, China; ^2^School of Mathematical Sciences, University of Electronic Science and Technology of China, Chengdu, China; ^3^Exercise and Mental Health Laboratory, School of Psychology, Shenzhen University, Shenzhen, China; ^4^Information Technology Center, Chengdu Sport University, Chengdu, China; ^5^School of Public Affairs and Administration, University of Electronic Science and Technology of China, Chengdu, China; ^6^Ministry of Education Key Lab for Neuroinformation, High-Field Magnetic Resonance Brain Imaging Key Laboratory of Sichuan Province, University of Electronic Science and Technology of China, Chengdu, China; ^7^The Clinical Hospital of Chengdu Brain Science Institute, School of Life Sciences and Technology, University of Electronic Science and Technology of China, Chengdu, China

**Keywords:** athlete training, global functional connectivity density, resting-state functional magnetic resonance imaging, attention, neuroplasticity

## Abstract

The human brain shows neuroplastic adaptations caused by motor skill training. Of note, there is little known about the plastic architecture of the whole-brain network in resting state. The purpose of the present study was to detect how motor training affected the density distribution of whole-brain resting-state functional connectivity (FC). Resting-state functional magnetic resonance imaging data was assessed based on a comparison of fast-ball student athletes (SA) and non-athlete healthy controls (NC). The voxel-wise data-driven graph theory approach, global functional connectivity density (gFCD) mapping, was applied. Results showed that the SA group exhibited significantly decreased gFCD in brain regions centered at the left triangular part of the inferior frontal gyrus (IFG), extending to the opercular part of the left IFG and middle frontal gyrus compared to the NC group. In addition, findings suggested the idea of an increased neural efficiency of athletes’ brain regions associated with attentional–motor modulation and executive control. Furthermore, behavioral results showed that in the SA group, faster executive control reaction time relates to smaller gFCD values in the left IFG. These findings suggested that the motor training would decrease the numbers of FC in IFG to accelerate the executive control with high attentional demands and enable SA to rapidly focus the attention to detect the intriguing target.

## Introduction

The brain of skilled elite athletes who have improved sport performance reveals neuroplastic adaptations caused by motor skill training (Gao et al., 2019; Chen et al., 2020; Jung et al., 2020). It has attracted special attention from researchers exploring how and where the structural and functional plasticity of the brain occurs in the course of motor skill training using imaging techniques [e.g., functional magnetic resonance imaging (fMRI) and functional near infrared spectroscopy (fNIRs)] (Crawford et al., 2019; Yu et al., 2020; Yue et al., 2020a, b). In particular, fMRI is widely accepted as an effective method to help researchers to understand the physiological mechanisms of brain plasticity via motor training; furthermore, findings might be practically meaningful for improving athletic performance (Gao et al., 2019; Zhang et al., 2019). However, it is largely unknown how brain functions change in the process of motor training (Lappi, 2015). Some researchers attempted to investigate the difference in brain activity between athletes and non-athlete controls (NC) during certain tasks (Gao et al., 2019), with different regions of task-induced activation between the two groups that are being reported. For instance, in the visual smooth pursuit task, football players showed greater activation in the oculomotor region of the cerebellar vermis and areas of the frontal eye fields than NC, suggesting visual motor skill is required for elite football players to successfully execute complex movement during games (Kellar et al., 2018). In addition, enhanced inferior parietal/frontal gyrus activity has been proposed in basketball players anticipating a free-throw task (Wu et al., 2013). Previous research suggested that when familiar sports environmental sounds surrounded, elite athletes tend to have greater activation in somatosensory regions but less activation in brain regions, which associated with perception and motor planning and processing (Woods et al., 2014). Except for the aforementioned results, a decreased brain activation in athletes (e.g., archery and table tennis) was reported across various cognitive tasks (e.g., mental rehearsal and visual–spatial task) compared to non-experts (Chang et al., 2011; Guo et al., 2017).

Resting-state fMRI (rsfMRI) is the commonly used method in the human brain map to evaluate intrinsic spontaneous fluctuations in the blood oxygen level-dependent (BOLD) signal in the resting or task-free state (Gao et al., 2013). This innovative and effective method was widely used in previous studies to investigate intrinsic brain plasticity or regional interaction induced by motor training. A study which implemented seed-based functional connectivity (FC) in distance runners shows increased connectivity between the fronto-parietal network and brain regions for execution functions compared to non-expert runners (Raichlen et al., 2016). These functional alterations in fronto-parietal connectivity were also found in badminton athletes (Di et al., 2012; Xu et al., 2016). In addition, similar results are found in elite karate players, suggesting brain areas associated with movement planning and visual perception having increased connectivity (Duru and Balcioglu, 2018). Our recent study using seed-based stepwise FC showed that fast-ball athletes were reported with a significantly smaller optimal connectivity distance from seed regions to the dorsal attention network (DAN) and larger optimal connectivity distance to the default mode network (DMN; Gao et al., 2019). Given the evidences above, changes in resting-state-FC brain plasticity occur in different brain areas across various sport events.

However, seed-based methods involved with the *a priori* selection of appropriate seed regions are unable to fully characterize the brain functional connectome as well as get a whole picture on the plastic architecture of the whole-brain network (Joel et al., 2011). Recently, functional connectivity density (FCD) mapping, a voxel-wise data-driven graph theory approach, has been established to determine the density distribution of whole-brain resting-state FC (Tomasi and Volkow, 2011; Huang et al., 2018). It measures the number of functional connections of a given voxel with the remaining voxels in the whole brain. The brain regions with high FCD values are considered as functional hubs of the human brain, which play a very important role in brain function (Tomasi and Volkow, 2011). Thus, this approach is a reliable and effective method and has been increasingly accepted in research detecting biomarkers of neuropsychiatric disorders through resting-state functional network alterations, including schizophrenia (Huang et al., 2018), major depressive disorder (Zhang et al., 2016; Zou et al., 2016), migraine (Gao et al., 2016), and aging brain (Li et al., 2019).

Here, we aimed to explore the neuroplasticity on brain functional organization induced by motor training using the global FCD (gFCD) approach. Fast-ball student athletes (SA) who play badminton, tennis, and table tennis were recruited. In fast-ball sports like badminton, tennis, and table tennis, the players require well-refined hand–eye coordination and visuospatial ability to achieve improved sports-related cognitions such as perception, focus, anticipation, planning, and fast responses (Di et al., 2012; Wolf et al., 2014). We started with an assumption that the fast-ball athletes would show changes in FC architecture in brain regions related to the visual attention and visual–motor coordination. To test whether the brain gFCD values relate to attentional processes, all subjects were asked to perform a revised attention network test (ANT) processing. The relationships between gFCD and reaction time of each attention subnetwork of alerting, orienting, and executive control were also investigated.

## Materials and Methods

### Participants

Forty-two SA majoring in fast-ball sports (badminton, tennis, and table tennis) were recruited from Chengdu Sport University. All SA were required to pass the college entrance examination for sports majors in Sichuan province of China before entering Chengdu Sport University. The examination consisted of 40 points of the sport-specific test and 20 points of three physical fitness events, including 100-m run, standing triple jump, and standing shot put. The full mark of all test items is based on or slightly higher than the Standard of Technical Grade of Athletes issued by the General Administration of Sport of China. For example, the full mark of 100 m is 11.54 s, and the total score of all four test items has to exceed 85 points. After entering Chengdu Sport University, SA have been engaged in the fast ball-specific training. The training duration was less than 3 years, and the training frequency was less than 25 h per week. Thirty-nine NC with matched age, gender, and education level were also recruited. Except the sports-required course in the university, the NC group spent no more time on physical activity. All participants were tested handedness using the Chinese version of the Edinburgh-Handedness Questionnaire (coefficients > 50) (Oldfield, 1971), and all showed right-handedness. Participants had no history of neurological or psychiatric diseases or concussions. The study protocol was approved by the research ethical committee of School of Life Sciences and Technology, University of Electronic Science and Technology of China. Participants were provided written informed consent prior to any assessment.

### Data Acquisition

Magnetic resonance imaging (MRI) images were acquired on a 3.0 T GE Signa MR750 system (GE Healthcare, Milwaukee) with an 8-channel phased array head coil. High-resolution 3D T1-weighted anatomical images were obtained in axial orientation using a 3D spoiled gradient-recalled (SPGR) sequence. The acquisition parameters were as follows: TR = 5.97 ms, TE = 1.96 ms, field of view (FOV) = 240 mm × 240 mm, flip angle = 12°, matrix size = 512 × 512, 156 slices, and voxel size = 1 mm × 1 mm × 1 mm. Resting-state fMRI images were acquired using a gradient-recalled echo planar imaging (EPI) sequence. The parameters were TR = 2000 ms, TE = 30 ms, FOV = 220 mm × 220 mm, flip angle = 90°, matrix size = 64 × 64, 43 transverse slices without a slice gap, voxel size = 3.75 mm × 3.75 mm × 3.2 mm, and a total of 266 volumes for each subject. During the scan, the subjects were instructed to lie down with their eyes closed, not to think of anything in particular, and not to fall asleep. Padded foams were used to restrict head motion, and earplugs were used to attenuate scanner noise.

### Data Preprocessing

Conventional fMRI data preprocessing was performed using Data Processing Assistant for Resting-State fMRI software (DPARSF, Advanced Edition, V4.5).^1^ The first 10 volumes of each subject were discarded to ensure steady-state longitudinal magnetization. The remaining 256 resting-state fMRI images were first corrected for the acquisition time delay between different slices and then realigned to the first volume to correct for head motion. We required that the transient movement during the scanning was no more than 1.0 mm of translation and 1.0° of rotation. The images were further spatially normalized into a standard stereotaxic space at 3 mm × 3 mm × 3 mm, using the EPI template in the Statistical Parametric Mapping software (SPM8). The images were not smoothed to avoid introducing artificial local spatial correlations. Images were then linearly detrended and were corrected using linear regression to remove the possible spurious variances including 24 head-motion parameters, averaged signals from cerebrospinal fluid, and white matter. The residuals of these regressions were temporally band-pass filtered (0.01 < *f* < 0.08 Hz) to reduce low-frequency drifts and physiological high-frequency respiratory and cardiac noises. Finally, scrubbing with the interpolation method was used to remove the bad points of the data. Since FC analysis is sensitive to gross head motion effects (Power et al., 2012), the mean frame-wise displacement (FD) was calculated to further determine the comparability of head movement across groups. The largest FD obtained from the subjects was less than 0.2 mm (which was 0.178 mm).

### Global Functional Connectivity Density Analysis

Global functional connectivity density is defined as the number of statistically significant FCs between a given voxel and the rest of voxels across the whole brain in a binary network (Tomasi and Volkow, 2010). It is capable of quantifying the importance/centrality of a given voxel within the whole-brain network (Tomasi and Volkow, 2010). The voxel-wise gFCD maps were computed by an in-house program coded in MATLAB (The MathWorks, Natick, MA) according to the approach introduced by Tomasi and Volkow (2010). Briefly, we calculated the Pearson’s correlation (*r*) between brain voxels that limited in a gray matter mask based on the automated anatomical labeling (AAL) atlas to obtain a whole-brain FC map at the voxel level. Two voxels were considered to be connected if their Pearson’s correlation coefficient of the two voxels was greater than a given correlation threshold *Tc* = 0.6 according to the significant level of *p* < 0.01 (Bonferroni multiple correction), in order to eliminate the weak correlation which may be caused by noise (Tomasi and Volkow, 2010; Huang et al., 2018). Here, the significant level was set for testing the hypothesis of no functional connection against the alternative that there is a functional connection (non-zero correlation). The value of gFCD was defined of the global number of functional connections *n*_*i*_, between voxel *i* and all other voxels in the brain. For each participant, gFCD maps were further standardized by the total number of edges in the whole brain (Li et al., 2018) and were spatially smoothed with full-width at half-maximum (FWHM) = 8 mm to minimize the individual overall differences of gFCD values (Tomasi and Volkow, 2011).

### The Attention Network Test

**FIGURE 1:**
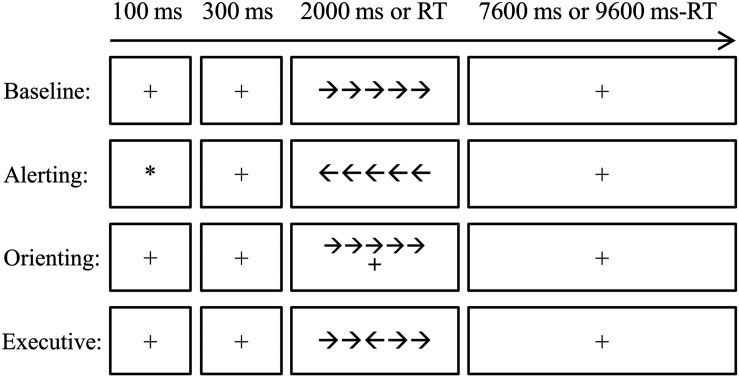
The diagram for the attention network test (ANT) procedure. The four conditions, i.e., baseline, alerting, orienting, and executive control, were arranged in separated blocks. Each trial started with a cue (the alerting condition) or a fixation with 100 ms. After a 300-ms interval, the target was presented for 2,000 ms or disappeared after one key was pressed. A fixation was then presented until the end of that trial. Each trial lasted for 10 s. RT, reaction time.

To test whether the altered FC network architecture relates to attentional processes, all subjects were asked to perform a revised ANT (Wang et al., 2016), as shown in Figure 1. Briefly, the stimuli were presented via E-Prime 2.0 (Psychology Software Tools, Inc^2^) on a Lenovo PC. Responses were collected via Q (for left targets) and P (for right targets) on the keyboard. All participants completed four blocks (alerting block, orienting block, executive control block, and baseline block) of the ANT. The order of the four blocks was counterbalanced across subjects. Each block took 424 s, which contained a buffer time of 4 s, two practice trials of 10 s, and 40 experimental trials of 10 s. As shown in Figure 1, each trial began with a fixation or cue for 100 ms which was followed by a 300-ms fixation. After that, a target (congruent or incongruent, central, or spatial) appeared for 2000 ms or until the participant pressed a key. Lastly, another fixation was presented to ensure that the overall time of one trial was 10,000 ms (0.1 Hz). The subjects were asked to judge the direction of the third arrow (the central one) by pressing Q if it points to the left and P if it points to the right. The attention network scores (ANSs) were computed to measure the reaction time of each attention subnetwork of alerting, orienting, and executive control compared to the baseline (Wang et al., 2016).

### Statistical Analysis

We used two-sample *t*-test analysis to compare the group differences of gFCD between the two groups. Age, gender, and education were considered as covariate variables. The statistically significant threshold of the gFCD was set for multiple comparisons at the cluster level with *p* < 0.05 (AlphaSim corrected). This correction was conducted using the DPARSF software.

To investigate whether the altered gFCD was associated with the behavioral reaction time of each attention subnetwork of alerting, orienting, and executive control, relationships between gFCD values in regions showing significant group differences and the reaction time of the three attention subnetworks were further detected by partial correlation analysis. The partial correlation analysis was performed for the SA group and the NC group, separately, with age and gender as confounding factors.

## Results

### Demographic Data

The demographic data of the recruited subjects are shown in Table 1. The athlete group and the control group did not differ significantly in age (Mann–Whitney U-test, *p* = 0.17), gender (Pearson χ^2^ test, *p* = 0.35), education (Mann–Whitney U-test, *p* = 0.58), or mean FD (Mann–Whitney U-test, *p* = 0.94).

**TABLE 1:** Demographics of the subjects.

	Age (years)	Gender (female/male)	Education (years)	Training time (h/week)	Duration (years)	Mean FD (mm)
SA (*n* = 42)	20.43 ± 0.59	12/30	14.57 ± 0.67	17.17 ± 0.83	1.95 ± 0.08	0.08 ± 0.02
NC (*n* = 39)	20.33 ± 1.33	15/24	15.05 ± 1.43	–	–	0.08 ± 0.03
*P* value	0.1736^*a*^	0.3454^*b*^	0.5803^*a*^	–	–	0.9363^*a*^

*Data are presented as mean ± SD. SD, standard deviation; FD, frame-wise displacement; NC, non-athlete controls; SA, student athlete. ^*a*^Obtained using Mann–Whitney U-test. ^*b*^Obtained using the Pearson χ^2^ test.*

### gFCD in Athletes and Controls

**FIGURE 2:**
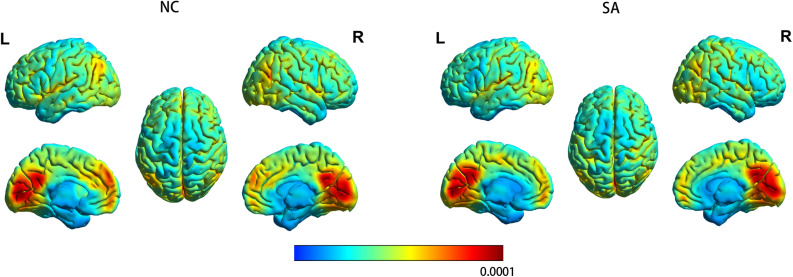
The spatial distribution maps of the average gFCD maps for the SA group and the NC group. For each participant, the gFCD value of each voxel was standardized by the total number of edges in the whole brain. L, left; NC, non-athlete control; R, right; SA, student athlete.

**FIGURE 3:**
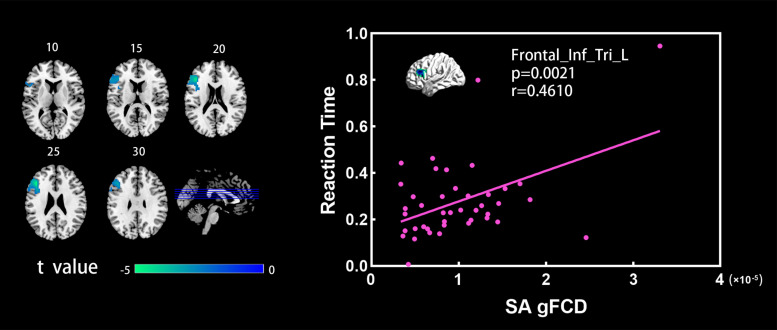
**(Left)** Brain regions showing significant differences between the SA and NC groups in the gFCD analysis (two sample *t*-test, *p* < 0.05, GRF corrected). Numbers above each image refer to the *z*-plane coordinates of the MNI space. **(Right)** Significant correlation between reaction time and gFCD values in the SA group. Frontal_Inf_Tri_L, left triangular part of inferior frontal gyrus.

Figure 2 shows the spatial distribution maps of the average gFCD in the SA group and the NC group, respectively. In both the SA and NC groups, the prefrontal cortex, posterior cingulate cortex, precuneus, and occipital cortex had relatively high gFCD values, as reported in previous studies (Tomasi and Volkow, 2010; Luo et al., 2014; Huang et al., 2018). Figure 3 (left) shows the significantly different gFCD between the groups (*p* < 0.05, AlphaSim corrected). The SA group exhibited significantly decreased gFCD in brain regions centered at the left triangular part of the inferior frontal gyrus (IFG), extending to the left opercular part of the IFG and middle frontal gyrus (MFG) compared to the NC group. Table 2 presented the Montreal Neurological Institute (MNI) coordinates of peak voxels and statistical *t*-values of the brain regions with significantly different gFCD between the two groups. The positive *t*-values represented that the NC group had higher gFCD values than the SA group. No significantly increased gFCD in the SA group compared with the NC group was found.

**TABLE 2:** Brain regions showed significantly different gFCD between the athlete group and the control group.

Brain regions	Hem	Peak coordinates	Cluster size	*T*-value
Inferior frontal gyrus, triangular	L	(−39,27,24)	328	5.63
Inferior frontal gyrus, opercular	L	(−57,27,18)	68	3.83
Middle frontal gyrus	L	(−54,36,21)	144	3.72

### ANT Scores and Relationships With gFCD

Figure 3 (right) shows the significant correlations between the gFCD values and the reaction time of executive control in the SA group. In the SA group, the gFCD values in the left triangular part of IFG showed significant positive correlations with the executive control reaction time. In the NC group, subjects had relatively low values in reaction time, and the within-group dispersion of the reaction time was relatively small. The significant correlation between the gFCD values and the reaction time was not detected in the NC group.

## Discussion

The frontal cortex is one of the most crucial areas related to high-level functional integration (Woods et al., 2014). The inferior and middle frontal cortex especially contributed to the visual attention modulation and executive functions (Woods et al., 2014; Guo et al., 2017). In the task research in athletes, expert athletes had less activation in IFG and MFG when listening to familiar sports environmental sounds, suggesting the neural efficiency in these brain regions associated with perception and motor planning and processing (Woods et al., 2014). During the motor reaction and the visuo-spatial tasks, formula racing-car drivers recruited distributed networks including the middle and inferior frontal cortices, which devoted to executive functions (Bernardi et al., 2013). However, professional drivers recruited these regions to a significantly smaller extent as compared to naïve subjects (Bernardi et al., 2013). As support, table tennis athletes perform the visuo-spatial task with less brain activation in bilateral MFG than non-athletes (Guo et al., 2017). A similar finding was also observed in expert hockey players, suggesting neural efficiency in these brain regions for action decision as they were presented videos for shooting a puck toward a hockey goal (Olsson and Lundstrom, 2013). However, there were controversial results in some tasks. For instance, an increased activity in the inferior parietal lobule and IFG was detected in basketball athletes performing a free-throw task (Wu et al., 2013). Therefore, the task-related results demonstrated IFG and MFG as functional hubs of visual motor integration, motor control, and executive functions. In addition, the results suggested their functional plasticity induced by motor skill training.

The rsfMRI results are independent of task. The task-free approach can explore the intrinsic neural plasticity induced by motor skill training. There has been local seed-based FC analysis, which showed altered FC between the athlete group and controls within the left frontoparietal network (Di et al., 2012). In the athlete group, an enhanced FC between the left superior parietal lobule and left MFG (BA6) was detected, whereas a lower FC between the left superior parietal lobule and the left MFG (BA9) was observed compared to the control group (Di et al., 2012). In addition, previous research was found that distance runners exhibited enhanced connectivity between the right parietal cortex and MFG (Raichlen et al., 2016).

Global functional connectivity density is a powerful framework to characterize the FC architecture within the whole-brain network without any prior hypothesis (Tomasi and Volkow, 2011). From the point of view of the whole-brain resting-state FC, our results showed decreased gFCD values in the left IFG and MFG in the fast-ball SA group compared to the NC group. This is a distinct but complementary information on the brain plasticity caused by motor skill training in fast-ball SA. Such results demonstrated reduced connections between these brain regions and the remaining brain voxels in the SA group. The findings were consistent with the previous research, regardless of skilled groups, such as racing-car drivers (Bernardi et al., 2013), table tennis players (Guo et al., 2017), basketball players (Pi et al., 2019), golfers (Milton et al., 2007), and musicians (Haslinger et al., 2004; Meister et al., 2005). The possible explanation could be that athletes’ brain might exhibit an improved neural efficiency in the brain regions associated with attentional-motor modulation and executive control along with a reduced “resource consumption” (Rypma and Prabhakaran, 2009; Bernardi et al., 2013; Guo et al., 2017; Sommer et al., 2018; Zhang et al., 2019).

Importantly, we further detected a positive correlation between gFCD values in the left triangular part of IFG and reaction time of the executive control in the SA group. Of the three attention subnetworks, the executive control network is very useful for producing top-down regulation and thus is related to executive control (Petersen and Posner, 2012). This system was presented under the heading of target detection (Petersen and Posner, 2012). It is related to the limited capacity of the attention system, and to awareness itself, and has often been called focal attention (Petersen and Posner, 2012). Fast-ball sport events require athletes to execute actions in a short time (Ermutlu et al., 2015), in terms of well-refined hand–eye coordination and visuospatial ability to achieve enhanced perception, concentration, anticipation, planning, and fast response requirements (Di et al., 2012; Gao et al., 2019). Our results suggested that faster executive control reaction time was associated with smaller gFCD values in the left triangular part of IFG. The behavioral results further supported that in the SA group, shorter reaction time of executive control was associated with smaller gFCD values, which referred to the numbers of FC between a given voxel and the rest of voxels across the whole brain. The findings implied that motor skill training results in a decrease in the number of FC in the left triangular part of IFG, which athletes are interested in developing reaction capacity, movement planning, and execution with high attentional demands, and focus the attention to the target detection (Wolf et al., 2014). Of note, sample size was relatively low in the present study, and only the student-athletes who were trained in fast ball of badminton, tennis, and table tennis were recruited. Our findings only limited to the fast-ball sports athletes with early stage of motor training.

## Conclusion

Using resting-state functional imaging techniques and graph theory approach, the present study revealed that compared with the NC group, the SA group exhibited significantly decreased gFCD in IFG and MFG. As supportive of the behavioral data, faster executive control reaction time was associated with smaller gFCD values in the left triangular part of IFG in the SA group. The findings supported the idea of an increased neural efficiency of athletes’ brain in the brain regions associated with attentional-motor modulation and executive control and implied the training-induced neuroplasticity occurring in these brain regions. In addition, our findings were based on the resting-state brain functional network, which was independent of specific tasks. The neural mechanisms of functional adaptations in the athletes’ brain that made their exceptional performance possible might have potential applications in designing optimal sports coaching methods, in overcoming learning disabilities, and in neurological rehabilitation (Lappi, 2015).

## Data Availability Statement

The datasets generated for this study are available on request to the corresponding author.

## Ethics Statement

The study protocol was approved by the research ethical committee of School of Life Sciences and Technology, University of Electronic Science and Technology of China. The patients/participants provided their written informed consent to participate in this study. Written informed consent was obtained from the individual(s) for the publication of any potentially identifiable images or data included in this article.

## Author Contributions

CY, NL, and QG contributed to the conception and design of the research. MZ, JG, HW, and ML collected the data. ML, SZ, and JZ applied the statistical analysis. NL and CY analyzed and interpreted the data. CY, NL, and QG wrote the manuscript. QG and HC edited the manuscript. QG was responsible for the overall project. QY edited and modified the manuscript. All authors contributed to the article and approved the submitted version.

## Conflict of Interest

The authors declare that the research was conducted in the absence of any commercial or financial relationships that could be construed as a potential conflict of interest.

## Footnotes

1 http://www.restfmri.net/forum/

2 http://www.pstnet.com
